# Health and saliva microbiomes of a semi-urbanized indigenous tribe in Peninsular Malaysia

**DOI:** 10.12688/f1000research.17706.3

**Published:** 2019-05-28

**Authors:** Li-Fang Yeo, Farhang F. Aghakhanian, James S. Y. Tan, Han Ming Gan, Maude E. Phipps

**Affiliations:** 1 Jeffrey Cheah School of Medicine and Health Sciences, Monash University, Bandar Sunway, Selangor, 46150, Malaysia; 2 Tropical Medicine and Biology Platform, Monash University, Bandar Sunway, Selangor, 46150, Malaysia; 3 Imperial College London, South Kensington Campus, London, SW7 2AZ, UK; 4 School of Life & Env Sciences, Deakin University, Geelong Waurn Ponds Campus, Australia

**Keywords:** Orang Asli, saliva microbiome, anthropometrics, cardio-metabolic health, indigenous people

## Abstract

**Background:** The indigenous people of Peninsular Malaysia, also known as Orang Asli, have gradually been urbanized. A shift towards non-communicable diseases commonly associated with sedentary lifestyles have been reported in many tribes. This study engaged with a semi-urbanized Temiar tribe from Kampong Pos Piah, Perak, who are experiencing an epidemiological transition.

**Methods:**  Weight, height, waist circumference, blood pressure, HbA1C and lipid levels were measured as indicators of cardio-metabolic health. DNA was extracted from saliva using salting-out method followed by PCR amplification of the V3-V4 region of the 16S rRNA gene and sequencing on Illumina MiSeq. Microbiome analysis was conducted on Qiime v1.9. Statistical analysis was conducted using Qiime v1.9 and R.

**Results:** The study revealed that 60.4% of the Temiar community were overweight/obese, with a higher prevalence among women. HbA1C levels showed that 45% of Temiar had pre-diabetes. Insulin resistance was identified in 21% of Temiar by using a surrogate marker, TG/HDL. In total, 56.5% of Temiar were pre-hypertensive, and the condition was prevalent across all age-groups. The saliva microbiome profiles of Temiar revealed significant differences by gender, BMI, abdominal obesity as well as smoking status. The relative abundance of the genus *Bifidobacterium* was increased in men whereas the genera *Prevotella*, *Capnocytophaga, Leptotrichia, Neisseria and Streptococcus *were increased in women. Proteobacteria was significantly depleted in smokers.

**Conclusions:** Temiar from Pos Piah had a high prevalence of cardio-metabolic risks, including general and abdominal obesity, pre-diabetes, prehypertension and hypertension. This phenomenon has not been previously reported in this tribe. The saliva microbiome profiles were significantly different for individuals of different gender, BMI, abdominal obesity and smoking status.

## Introduction

The Orang Asli (OA), which means “original people” in the Malay language, comprise approximately 0.5% (150,000) of the total Malaysian population ^1^. They are categorized into three main groups, namely Negrito, Senoi and Proto Malay. OAs are widely spread across the Peninsular and range from semi-nomadic deep forest hunter-gatherers such as the Jahai to resettled communities such as Mah Meri to urbanized city-fringe dwellers such as Orang Seletar ^1^. This study focused on the Temiar who are a subtribe of Senoi and are believed to be descendants of the first Neolithic farmers who migrated to the Malay Peninsula ^2^.

In recent years, many OA communities were resettled by the government in effort to improve their lives. As the OA became more urbanized and by large left their ancestral habitats and practices, they led more sedentary lifestyles. These factors, coupled with loss of access to forest resources and increasing pressures to turn to store-bought food, may largely explain the rise in cardio-metabolic diseases such as hypertension, diabetes and obesity shown in recent studies ^1, 3, 4^.

The launch of the Human Microbiome Project heralded the unprecedented investigations of various microbiomes. Of these, oral microbiomes had been widely studied in human health and diseases ^5, 6^. Studies implied an oral origin to systemic diseases such as cardiovascular diseases as the oral cavity is a major gateway into the body ^7, 8^. Studies also investigated associations between oral microbiome and diabetes ^9^, and obesity ^10^, with mixed results. There were suggestions that obese people may have a different salivary bacterial composition perhaps akin to inflammation, which contributed to periodontal diseases and caries ^11^.

Little is known about the microbiomes of indigenous communities in Asia. To our knowledge, this was a pioneering investigation of their saliva microbiomes. Furthermore, biomedical studies of Temiar were sparse and outdated, despite them being a very large community. With this impetus, our study aimed to address the gap in knowledge by reporting on the anthropometrics and cardio-metabolic health of a resettled Temiar community and investigated their saliva microbiome in association with their health.

## Results

### Anthropometrics and cardio-metabolic health

A total of 72 Temiars, 33 men and 39 women, participated in the study. The median age was 34 years old. General and abdominal obesity had higher prevalence among Temiar women ( Table 1). Notably, 71.4% (n=25) of women and 28.1% (n=9) of men displayed abdominal obesity.

**Table 1.** Anthropometrics and cardio-metabolic risk factors among Temiar.

Variable	Whole, n (%)	Men, n (%)	Women, (%)
**General obesity (BMI)**			
Underweight (<18.5)	7 (10.3%)	4 (12.5%)	3 (8.3%)
Normal (18.5-22.9)	20 (29.4%)	12 (37.5%)	8 (22.2%)
Overweight (23-24.9)	15 (22.1%)	6 (18.8%)	9 (25%)
Pre-obese (25-29.9)	22 (32.4%)	10 (31.3%)	12 (33.3%)
Obese (≥30)	4 (5.9%)	0 (0%)	4 (11.1%)
**Abdominal obesity (waist circumference)**			
Healthy (M: <90cm; W: <80cm)	-	23 (71.9%)	10 (28.6%)
Risk (M: ≥90cm; W: ≥80cm)	-	9 (28.1%)	25 (71.4%)
**HbA1C level**			
Normal (4.0-5.6%)	36 (52.2%)	15 (46.9%)	21 (56.8%)
Pre-diabetes (5.7-6.4%)	31 (44.9%)	17 (53.1%)	14 (37.8%)
Diabetes	2 (2.9%)	0 (0%)	2 (5.4%)
**Insulin resistance (TG/HDL 0.9-1.7)**			
Normal	51 (78%)	20 (75%)	32 (94%)
Risk	14 (22%)	10 (15%)	4 (6%)
**Blood pressure**			
Normotensive	14 (20.3%)	7 (21.9%)	7 (18.9%)
Pre-hypertensive	39 (56.5%)	14 (43.8%)	24 (64.9%)
Stage 1	12 (17.4%)	9 (28.1%)	3 (8.1%)
Stage 2	4 (5.8%)	2 (6.3%)	3 (8.1%)

HbA1C levels indicated 44.9% of Temiar to be pre-diabetic, with a higher prevalence in men. The high prevalence of prediabetes is worrying because it indicates a rise in non-communicable diseases that was previously of low prevalence in rural communities. Using a TG/HDL as a surrogate marker for insulin resistance, 22% of Temiar were at risk of IR, mostly affecting men.

Blood pressure measurements showed that 56.5% (n=39) had pre-hypertension, which was more prevalent among women and was prevalent across all age groups. Stage 1 hypertension prevalence rate was 17.4% (n=12) and was found more prevalent among men. Raw data for these measurements are available in figshare ^12^.

### Saliva microbiome analysis

To analyse the saliva microbiota diversity, the V3-V4 hypervariable region on the 16S rRNA gene was amplified and sequenced on Illumina MiSeq. After data quality control (QC), a total of 991,006 reads with mean 14,362±78 reads per individual remained.

**Figure 1.**
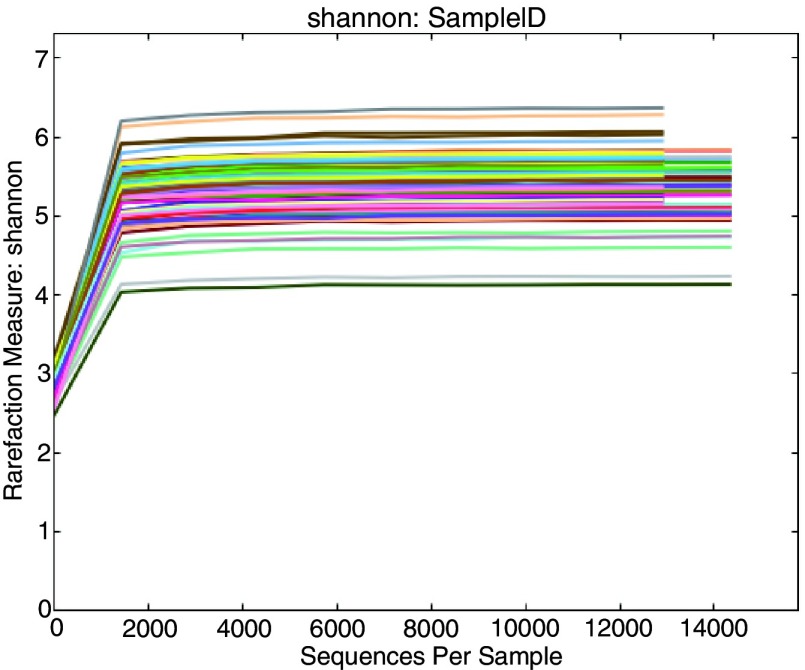
Rarefaction curve plotted using alpha diversity metric, Shannon index against number of sequences per sample. Each coloured line represents one biological sample.

**Figure 2.**
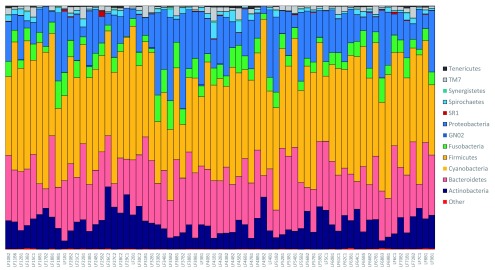
Relative abundance of bacteria found in the oral microbiome of Temiar at the phylum level.

To investigate whether the samples were sequenced to a sufficient depth, a rarefaction curve was plotted using the alpha diversity metric, Shannon index. Each colour represents a sample. The rarefaction curve indicated that all 69 samples were sequenced to a sufficient depth ( Figure 1). Reads were aligned to Greengenes database V13. The major OTUs (Operational Taxonomic Unit) at the phyla level observed include Actinobacteria, Bacteroidetes, Firmicutes, Fusobacteria and Proteobacteria ( Figure 2). These are the features of common oral microbiomes ^13^. OTUs shall henceforth be referred to as bacterial species for the ease of reading.

PERMANOVA was used to investigate the saliva microbiome compositions using UniFrac ^14^ distance matrix and were found to be associated with gender, obesity, waist circumference and smoking habits. To determine whether the relative abundance of individual bacterial species was differently represented in association with the factors investigated, we used the Kruskal-Wallis test with the OTU table as input.

**Figure 3.**
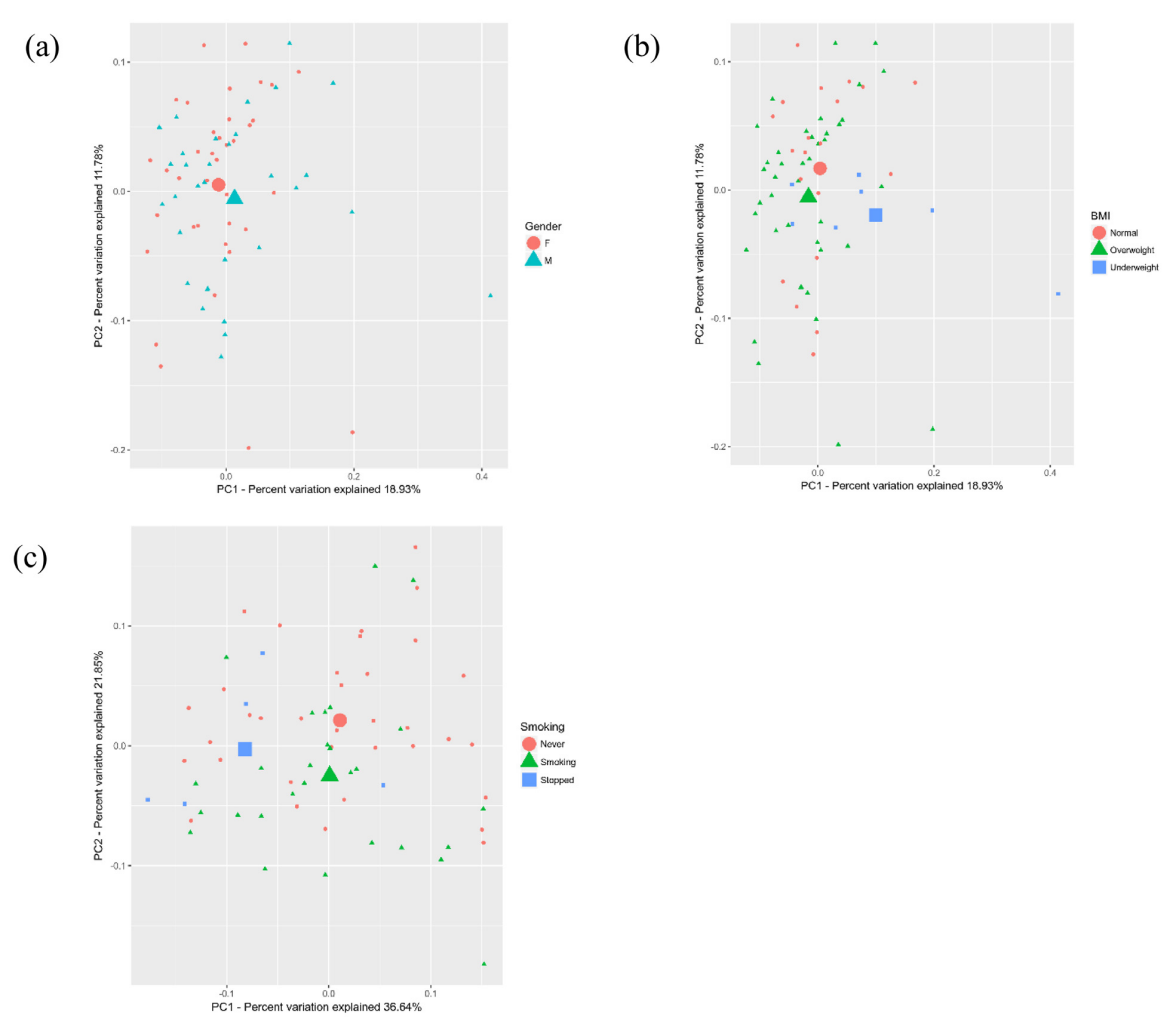
PCoA plots with the larger shapes representing the mean UniFrac distance between samples segregated by (a) gender – unweighted UniFrac; (b) body mass index (BMI) – unweighted UniFrac; (c) smoking status – weighted UniFrac.

Weighted UniFrac which takes into consideration the abundance of bacteria species revealed that the salivary bacteria were not significantly different between the two genders using PERMANOVA (p-value = 0.165, pseudo-F = 1.546, r ^2^=0.02). However, we found that the saliva microbiomes differed significantly between men and women for unweighted UniFrac (p-value = 0.028, pseudo-F = 1.824, r ^2^=0.02; Figure 3a). Unweighted UniFrac is a qualitative distance matrix that considers only the presence/absence of bacteria species. Kruskal-Wallis test revealed that the relative abundance of the genera *Prevotella*, *Capnocytophaga*, *Leptotrichia*, *Neisseria* and *Streptococcus* were significantly increased in women’s saliva microbiomes ( Table 2). These commensal oral bacteria may become opportunistic pathogens in immuno-compromised states ^8^. The relative abundance of the genus *Bifidobacterium*, was found to be highly elevated in men.

**Table 2.** Relative abundance of bacteria (genus level) differently represented in Temiar using Kruskal-Wallis test.

Bacteria (genus level)	FDR (q-value)	Abundance
*Prevotella*	0.006	More abundant in women
*Capnocytophaga*	0.021	
*Leptotrichia*	0.026	
*Neisseria*	0.026	
*Streptococcus*	0.036	
*Bifidobacterium*	0.043	More abundant in men

The saliva microbiome profiles also differed significantly with BMI (weighted UniFrac, p-value = 0.015, pseudo-F = 2.089, r2= 0.065; unweighted UniFrac, p-value = 0.029, pseudo-F = 1.989, r ^2^= 0.07; Figure 3b). A post-hoc Dunn’s test was conducted using the OTU table to test for differences between groups as PERMANOVA does not conduct pairwise comparison. The oral microbiome profiles of underweight individuals differed significantly from both overweight and normal individuals (underweight vs normal group, p-value = 0.0179; underweight vs overweight group, p-value = 0.0007). There was no significant difference between the saliva microbiome profiles of normal and overweight individuals (p-value = 0.0819). Differential abundance testing using Kruskal-Wallis test revealed that none of the bacterial taxa were significantly different.

There was a significant difference in the saliva microbiome of Temiar who had a healthy waist circumference compared to those with abdominal obesity (weighted UniFrac, p-value = 0.022, pseudo-F = 2.289, r ^2^ = 0.05). However, there was no significant difference in unweighted UniFrac (p-value = 0.286, pseudo-F = 1.099, r ^2^ = 0.021) as well as in individual bacterial taxa (p-value>0.05) among healthy individuals and those with abdominal obesity. The saliva microbiome composition of non-diabetic, pre-diabetic and diabetic individuals suggested some differences, but they were not significant (unweighted p-value = 0.069, pseudo-F = 1.502, r ^2^ = 0.043; weighted p-value = 0.122, pseudo-F = 1.579, r ^2^ = 0.045). The saliva microbiome composition and relative abundance of specific bacterial species were not statistically different when categorised by age group, lipid levels nor blood pressure levels.

There was a perceptible difference in the saliva microbiomes and smoking habits using weighted UniFrac (p-value = 0.016, pseudo-F = 2.498, r ^2^ = 0.07; Figure 3c) but no difference was detected when using unweighted UniFrac (p-value = 0.059, pseudo-F = 1.475, r ^2^ = 0.04). Further testing with Kruskal-Wallis showed the relative abundance of Proteobacteria and Firmicutes (phylum level) were significantly different among smokers and non-smokers. Differential abundance testing at the genus level revealed the relative abundance of *Neisseria* and *Aggregatibacter* was decreased in smokers compared to never-smokers and former smokers. Current smokers had a lower abundance of the genus *Neisseria* and *Aggregatibacter* than former smokers, but the difference was not statistically significant (p-value>0.05). The relative abundance of the genus *Campylobacter* and the class Clostridia, were higher in both current and former smokers compared to never-smokers ( Table 3).

**Table 3.** Relative abundance of bacteria found among smokers vs former and never-smokers using Kruskal-Wallis test.

Bacteria species		FDR(q-value)	Relative abundance of bacteria
Genus	*Neisseria*	0.025	Depleted in smokers and former smokers
	*Aggregatibacter*	0.035	Depleted in smokers and former smokers
	*Campylobacter*	0.037	Increased in smokers and former smokers
Class	Clostridia	0.037	Increased in smokers and former smokers

Overall, the relative abundance of the genera *Neisseria* and *Aggregatibacter* was decreased in current and former smokers, whereas the relative abundance of the genus *Campylobacter* and the class Clostridia was greater in smokers. The saliva microbiome showed no significant difference between former smokers and never-smokers

## Discussion

We reported a high prevalence of cardio-metabolic diseases such as obesity, pre-diabetes, insulin resistance and pre-hypertension among Temiar. These non-communicable diseases were previously not reported in OA ^15, 16^, but recent studies have indicated their high prevalence, especially in OA tribes living near cities ^1, 3, 17^. Increased cardio-metabolic risks were also reported in aboriginal Torres Straits Islanders from Australia ^18^, the Jaguapiru indigenous community in Brazil ^19^ and the Rang Bothiya tribe in India ^20^.

Many OA tribes lead relatively more sedentary lifestyles compared to their hunter-gatherer ancestors. They can no longer and perhaps have no need to rely entirely on the forest and its resources for survival. Rapid development had given them easier access to high-calorie processed foods, which may have contributed to obesity and cardio-metabolic diseases.

National Health and Morbidity Survey 2015 reported obesity prevalence among the major ethnic groups in Malaysia to be 30.6%, which is comparable to the findings in this study (Temiar obesity = 32.4%); the national prevalence rate of diabetes was reported to be 22.9% ^21^, which is very much higher than that of the Temiar (2.9%) in this study. Generally, reported prevalence rates of obesity among OA are still low. Nonetheless, the high prevalence rate of pre-diabetes reported among the Temiar (44.9%) indicates that rural OA communities are in dire need of awareness education and medical intervention.

Saliva microbiome analysis revealed significant difference in microbial composition among men and women, where the genus *Prevotella* was significantly higher in women. Hitherto, no studies have reported increased prevalence in women’s saliva microbiome. There are studies that suggest that hormones may play a role in the type of bacteria that colonise women’s oral cavity ^22– 26^ Temiar women had a lower prevalence of pre-diabetes and insulin resistance, despite the majority of them presenting with either general or abdominal obesity. Perhaps the women consumed a traditional, indigenous diet, which is richer in plant-fibre and less meat compared to men. Although *Prevotella* is a naturally occurring member of the oral microbiota ^27^, it is also associated with inflammatory conditions such as rheumatoid arthritis and periodontal infections ^28^.

The relative abundance of the genus *Bifidobacterium*, was shown to be elevated in Temiar men. While it is uncertain whether Temiar men were exposed to more dairy products than women, food taboos practiced among Temiar may contribute to the differences observed among gender ^29^. Several studies that investigated oral microbiomes of urbanized cohorts in association with gender have reported no differences in oral microbiome profiles ^23– 25^. This may be explained by the relatively homogenous environment that urbanized cohorts were exposed to. Studies have shown that salivary microbiomes are most affected by environmental factors, as the oral microbiome of twins which were similar became highly dissimilar when they lived apart ^23^.

Temiar, on the other hand, lived in a traditional environment where men and women had different social standings. Men went out to the forest to hunt or forage while women stayed in the village with the children. They also observed certain food taboos, where the bush meat consumption of animals such as river terrapin, gibbons and porcupine were reserved only for men ^29^.

This preliminary investigation suggested links between saliva microbiomes and gender where differences may be attributed to cultural, dietary and environmental factors. Even though the bacteria driving the differences in obesity and gender were of different species, it should be noted that most of the women were overweight/obese, which could be a confounding factor in gender-driven disparities in the saliva microbiome.

Studies have suggested an association between obesity and altered oral microbiome ^26, 30, 31^, concurring with the findings our study. However, a significant difference was noted only when comparing overweight and underweight individuals. Both states are considered to be ‘unhealthy’ and thus assumed to be at dysbiosis.

The oral hygiene practices and oral health among Temiar were unknown, although due to their geographical isolation, it was highly unlikely they have regular access to dental health care. Our study revealed that the relative abundance of the phylum Proteobacteria, and the genera *Neisseria* and *Aggregatibacter*, were decreased in smokers, compared to non-smokers. This was in line with the findings of Wu *et al.* (2016) ^32^, although our study did not detect differences in the several other genera reported by them. This was probably due to the differences in sample size of both studies.

Wu *et al.* (2016) reported that Proteobacteria were associated with the breakdown of toxic hydrocarbons found in cigarette smoke, hence a depletion of the bacteria genus in smokers may prove detrimental to oral health ^32^. An interesting difference noted was an increase in pathogenic anaerobe *Streptococcus* observed in their study ^32^, whereas Hernandez *et al.* (2017) ^33^ reported depletion of *Streptococcus* among betel-nut chewers, even after controlling for smoking. Our investigations revealed no significant difference, even though many of the Temiar smokers were also self-reported betel-nut chewers, a practice frequently associated with oral cancer ^34^. Further investigations may be required to distinguish the effects of betel-nut chewing and smoking on the saliva microbiome.

## Conclusion

Our study revealed a high prevalence of cardio-metabolic diseases among the Temiar, including general and abdominal obesity, pre-diabetes and insulin resistance. Pre-hypertension was found highly prevalent across all age groups.

Interestingly, the saliva microbiome profiles were significantly different for gender where the relative abundance of the genera *Prevotella, Capnocytophaga, Leptotrichia, Neisseria*, *Streptococcus* and *Bifidobacterium* were concerned. Our study also noted a significant difference between the saliva microbiome compositions of underweight vs overweight and normal individuals. The oral microbiome was not significantly different among non-diabetic, pre-diabetic and diabetic individuals. The microbiome profiles differed significantly among smokers and non-smokers where further investigation showed that the phylum Proteobacteria were significantly decreased in smokers. Investigation towards other health parameters such as pre-diabetes were inconclusive.

The sample size was small but sufficient to illustrate the health problems that plague the Temiar, especially the high prevalence of obesity, pre-diabetes and pre-hypertension. It would be very useful if future studies could determine the factors that are contributing to these health problems. Could it be a lack of awareness, overly sedentary lifestyle or perhaps a genetic predisposition? Regardless, studies have shown time and again that the OA are facing health problems that are reversible if they are made aware of them and the taught the correct methods to improve their lifestyle.

## Methods

### Data and sample collection

The study was approved by Ministry of Health Malaysia under National Medical Research Registry, MNDR ID #09—23-3913, Department of Orang Asli Development, Malaysia (JAKOA) and Monash University Human Research Ethics Committee (MUHREC). Before sampling, a courtesy visit to the Temiar elders in Kampong Pos Piah, Perak was conducted to explain the rationale of the study. Upon agreement to participate in our study, a medical team returned to the village on an agreed date and conducted health screening and sampling. Participants who were over 18 years old with no visible health ailments and able to provide informed consent were recruited for the study through convenience sampling, that is whoever who turned up and was eligible. Participants who were pregnant, with a history of alcohol/drug abuse, or with chronic illness (e.g. kidney failure, cancer, heart disease) were excluded from the study.

The consent form was read aloud by interviewers and queries were addressed before either a signature or thumbprint was provided as a sign of consent. A total of 72 Temiar provided informed consent to participate. Interviews were conducted in Bahasa Malaysia using a questionnaire ^12^ to collect information about their socio-demography, medical history and diet. Height, weight, waist circumference and blood pressure were measured ^1^. Participants were also examined by clinicians. Acanthosis negricans, which is darkening of the skin around the neck and creases of elbows indicative of insulin resistance, was noted.

Saliva samples were collected in sterile 50ml polypropylene Falcon tube. Participants were requested to rinse their mouths with water thoroughly 30 minutes prior to collecting saliva. Venous blood samples were taken for biochemical analyses.

### Anthropometrics and Biochemical analysis

BMI, waist circumference and blood pressure cut-off values were in accordance to WHO recommendations ^35^. We measured their HbA1C and blood lipid levels (cholesterol, HDL, LDL, Triglyceride). We used TG/HDL ratio as a surrogate marker for insulin resistance with a cut-off value of 0.9-1.7 ^36^.

### DNA extraction and PCR

DNA was extracted from saliva using a modified high salt-method ^37^. The V3-V4 region of the 16S rRNA gene were targeted, resulting in a PCR product of approximately 550 bp ^38^.

### Sequencing on Illumina MiSeq

DNA sequencing was done by Genomics Facility in Monash University Malaysia on Illumina MiSeq to produce paired end reads of approximately 230 bp each.

### Data analysis

Microbiome analysis was conducted on QIIME 1.9 ^39^. Chimeras were filtered using UCHIME 1.39.3 ^40^ before being aligned to Greengenes database V13.8 ^41^. The reads were then clustered into operational taxonomic units (OTUs) with open-reference method at 97% similarity level using UCLUST ^42^ in the QIIME pipeline. OTU clusters were assigned taxonomy with RDP classifier ^43^. The reads were normalized and OTUs that were present at less than 0.05% were filtered off.

Alpha diversity and beta diversity of the samples were reported using phylogenetic distance (PD) and UniFrac ^14^, respectively. PCoA plots were generated to visualize beta diversity of the samples.

### Statistical analysis

Statistical analyses were completed on QIIME and R 3.4.4. Information taken from the mapping file included gender, BMI and smoking status. PERMANOVA, a non-parametric test was used to test for differences in median among the groups using weighted and unweighted UniFrac distance matrix Usi//ng the R packages vegan (v2.4-2), readr (v1.1.0) and dplyr (v0.5.0). Kruskal-Wallis test was used to test for differences in the relative abundance of OTUs among the different groups. A post-hoc test, Dunn’s test was done for pairwise comparison when testing factors like BMI and smoking, as they had more than two groups. False discovery rate, reported as q-value, was used to control for multiple hypothesis testing and was statistically significant at 5%.

## Data availability

### Underlying data

Saliva microbiomes of the individuals in this study are available from the Sequence Read Archive, BioProject accession number PRJNA515166; https://identifiers.org/bioproject/PRJNA515166.

Anthropometric data, along with the other variables measured, as well as supplementary data are available on figshare. DOI: https://doi.org/10.26180/5c453ff435883 ^12^.

### Extended data

The questionnaire used in this study is available on figshare. DOI: https://doi.org/10.26180/5c453ff435883 ^12^.

Data are available under the terms of the Creative Commons Zero "No rights reserved" data waiver (CC0 1.0 Public domain dedication).
