# Supplementary material for: Health and saliva microbiomes of a semi-urbanized indigenous tribe in Peninsular Malaysia
Source: F1000Res. 2019 May 28;8:175. Originally published 2019 Feb 11. [Version 3] doi: 10.12688/f1000research.17706.3 (PMC6544134; doi:10.12688/f1000research.17706.3)
Supplement: Peer review history

# Peer review: Reviewer response for version 3

**DOI:** 10.5256/f1000research.21321.r49127

**Published:** 2019-05-29

**Authors:** Andres Gomez

**Affiliations:** 1 Department of Animal Science, University of Minnesota, Saint Paul, MN, USA

Thanks to the authors for addressing all the pending issues.

I have read this submission. I believe that I have an appropriate level of expertise to confirm that it is of an acceptable scientific standard.

# Peer review: Reviewer response for version 2

**DOI:** 10.5256/f1000research.21016.r48249

**Published:** 2019-05-22

**Authors:** Andres Gomez

**Affiliations:** 1 Department of Animal Science, University of Minnesota, Saint Paul, MN, USA

Figure 1. Please specify what each color on the rarefaction curve represents.

 Figure 3 and distance based analyses: Authors still have not followed through with adding R2 and pseudo-F values, besides P-values, in the text. I know it is referenced in the supplement (which by the way should be referenced throughout the text with specific S-table or S-figure number), but these three values should be referenced in the main text. Also, authors should specify in the text that the P-values from the distance-based differences calculated (also R2 and pseudo-F) were calculated via PERMANOVA.

 Also on figure 3, please omit "...representing the mean of distance matrix for.." It should read "representing the mean UniFrac distance between samples"

 PERMANOVA analyses: Authors have a confusion about the difference between then terms ADONIS and PERMANOVA- basically ADONIS, is a function within the R package vegan to perform PERMANOVA- the R2 is not a value from adonis, it's a value generated in PERMANOVA (all, P-values, R2 and pseudo-F). 

 This reviewer thanks the authors for improving the manuscript. However, before it can finally be endorsed, authors should address the following minor comments:

 Table 3: This is a comment made in the previous version: the title "Relative abundance of bacteria found among smokers vs former and never-smokers generated by QIIME" is incorrect - QIIME is am interface with several plugins, authors should specify which of these specific plugins allowed them to detect discriminant taxa in table 3 (e.g. LeFSE, binomial distributions?). Authors should specify I the text that discriminant taxa analyses were made at the genus level. Last, if authors mix the term OTU with several taxonomic levels (e.g. OTUS at the genius level, or OTUS at the phyla level, readers may get confused- typically, we only use OTUs as a proxy for species (>97% 16S rRNA sequence similarity).

I have read this submission. I believe that I have an appropriate level of expertise to confirm that it is of an acceptable scientific standard, however I have significant reservations, as outlined above.

# Peer review: Reviewer response for version 2

**DOI:** 10.5256/f1000research.21016.r48248

**Published:** 2019-05-17

**Authors:** Siti Nursheena Mohd Zain

**Affiliations:** 1 Institute of Biological Science, Faculty of Science, University of Malaya, Kuala Lumpur, Malaysia

The article reads well and all issues resolved.

I have read this submission. I believe that I have an appropriate level of expertise to confirm that it is of an acceptable scientific standard.

# Peer review: Reviewer response for version 1

**DOI:** 10.5256/f1000research.19359.r46035

**Published:** 2019-04-10

**Authors:** Siti Nursheena Mohd Zain

**Affiliations:** 1 Institute of Biological Science, Faculty of Science, University of Malaya, Kuala Lumpur, Malaysia

An interesting work looking at non-communicable disease risks link to microbiome in saliva as an indicator. The discussion could be written better to pull the results together. What is missing is a comparative study with another sedentary population. I would like if the conclusion is added with recommendations to improve the issue facing the Temiar tribe. Noted that sample size is small to make small but still warrants some finding to improve their health status.

I have read this submission. I believe that I have an appropriate level of expertise to confirm that it is of an acceptable scientific standard, however I have significant reservations, as outlined above.

# Peer review: Reviewer response for version 1

**DOI:** 10.5256/f1000research.19359.r44294

**Published:** 2019-03-13

**Authors:** Andres Gomez

**Affiliations:** 1 Department of Animal Science, University of Minnesota, Saint Paul, MN, USA

Results

 The QIIME microbiome analyses should be briefly described as far as the bioinformatics pipeline used and its details: clustering of OTUS? OTU picking? amplicon sequence variants (DADA2-ASV)? These should be specifically detailed.

 It does not seem that the rarefaction curve shows compete plateau, also what do the colors mean?

 PERMANOVA is used to mine for distinctions in microbiome composition.

 It is unclear how kruskal-wallis test was used in conjunction with Unifrac - KW is used for pairwise comparisons in multivariate space, not for multivariate data.

 PERMANOVA results (P values) should be accompanied by R2 and, Pseudo F values also. PERMANOVA models used should also be reported.

 "Kruskal-Wallis test revealed that the relative abundance of *Prevotella*, *Capnocytophaga*, *Leptotrichia*, *Neisseria* and *Streptococcus*​​​​​​"

 Were these discriminant analyses made at genus or OTU/ASV levels? Also, it is recommended to show these results in box plots showing medians and variation of data, not in tables 

 Authors make several assertions as to the character and typically reported nature of the markers discovered, in the context of "good/bad bacteria" - but many of the claims made are in the context of gut microbiomes not oral communities.

 For instance:

 "​​​​​​ *Prevotella*, associated with consumption of plant-rich diets"

 This claim may be true only for the gut microbiome not for oral communities. Also: "The relative abundance of Bifidobacterium, the ‘good bacteria’ commonly found in dairy products, was found to be highly elevated in men" - this may be associated with effect of dairy products in the gut not in the oral cavity. 

 "These commensal oral bacteria may become opportunistic pathogens in immune-compromised states." please cite

 Fig 3- legends should indicate that a-b were based on Unw-UniFrac, and that C on W-UniFrac - Please depict % variation on axes 

 It is not clear how the post-hoc Dun's test were conducted-0 is this still based on PERMANOVA?

 "The Kruskal-Wallis test generated by QIIME revealed no significant difference." This is not clear - compared to what? 

 "There was a significant difference between waist circumference and saliva microbiome composition (weighted UniFrac, p-value = 0.022). ​​​​​" This is not clear - you mean differences between the microbiomes of subjects with different waist circumference?

 QIIME is just a software with plugins to perform statistical analyses - it is not clear when authors are using qiime or R and how these platforms would yield different results. Please avoid jargon as "generated by QIIME"

 "The saliva microbiome showed no significant difference between former smokers and never-smokers, suggesting perhaps the saliva microbiome may recover partially to an environment prior to smoking". This is unclear but also speculative. 

 The discussion is highly speculative and should be toned down, mainly as far as the hypotheses behind the differences reported.

 Authors also seem to interchangeably extrapolate hypothesis on oral microbiome changes with gut microbiome changes in the context of lifestyle and diet (e.g. Prevotella) although diet may also be linked to oral microbiome modulation, this relationship is less clear, and taxa such as Prevotella, abundant in both gut and oral cavity, cannot be assumed to be modulated by diet in both sites

I have read this submission. I believe that I have an appropriate level of expertise to confirm that it is of an acceptable scientific standard, however I have significant reservations, as outlined above.

# Response

**Published:** 2019-03-18

**Authors:** Li-Fang Yeo

**Affiliations:** Monash University

Details on the bioinformatics pipeline will be addressed in version 2. The different colours on the rarefaction curve represent different individuals. Other metrics such as Shannon and Chao1 shows an obvious plateau. We felt that PD was more informative because it included phylogenetic distances.

 Our statistics were done mostly with reference to this paper:

 Wu, J., Peters, B.A., Dominianni, C., Zhang, Y., Pei, Z., Yang, L., Ma, Y., Purdue, M.P., Jacobs, E.J., Gapstur, S.M. and Li, H., 2016. “Cigarette smoking and the oral microbiome in a large study of American adults”. *The ISME journal*. https://www.nature.com/articles/ismej201637

 However, more recent methods such as using balances and log ratio shall be considered in a larger, extended study. Test scores for PERMANOVA results shall be addressed in version 2 as will several questions that were unclear in version 1.

 To date, we have none found literature that was comparable (ie. Having Bifidobacterium elevated in men, Prevotella elevated in women). Hence we attempted to suggest possible explanations to this observation. The discussion will be re-worded to take into account reviewer’s comments.

# Response

**Published:** 2019-05-20

**Authors:** Andres Gomez

**Affiliations:** University of Minnesota, USA

Dear authors,

Could you please provide point by point responses to the comments raised in the previous version, along with where changes were made in the manuscript?

That would facilitate reviewing of the new manuscript.

# Response

**Published:** 2019-05-20

**Authors:** Li-Fang Yeo

**Affiliations:** Monash University

Dear reviewer,

 As per request:

 1. The QIIME microbiome analyses should be briefly described as far as the bioinformatics pipeline used and its details: clustering of OTUS? OTU picking? amplicon sequence variants (DADA2-ASV)? These should be specifically detailed. 

**Microbiome analysis pipeline edited and detailed in Methods section.**

 2. It does not seem that the rarefaction curve shows compete plateau, also what do the colors mean?

**Rarefaction curve now shows Shannon index instead of phylogenetic diversity for better indicator of plateau. Each colour is a biological sample.**

 3. It is unclear how kruskal-wallis test was used in conjunction with Unifrac - KW is used for pairwise comparisons in multivariate space, not for multivariate data.

**KW test was conducted on an OTU table, not UniFrac. Refer to Ying & Sun (2017) **https://www.sciencedirect.com/science/article/pii/S2352304217300351

**Edits made in Results section.**

 4. PERMANOVA results (P values) should be accompanied by R2 and, Pseudo F values also. PERMANOVA models used should also be reported.

**Pseudo F values are reported. R2 value not available for PERMANOVA but available for adonis. All available values are reported in supplementary material.**

 5. ****"Kruskal-Wallis test revealed that the relative abundance of *Prevotella*, *Capnocytophaga*, *Leptotrichia*, *Neisseria* and *Streptococcus*​​​​​​"

 Were these discriminant analyses made at genus or OTU/ASV levels? Also, it is recommended to show these results in box plots showing medians and variation of data, not in tables 

**These analyses were made at OTU level 6, equivalent to genus level. Addressed in Results section. Also, due to time constraint and admittedly technical limitations on our part, the results will be shown in tables for this paper. Reviewer's advice are definitely well-received and will be considered for our next paper.**

 6. Authors make several assertions as to the character and typically reported nature of the markers discovered, in the context of "good/bad bacteria" - but many of the claims made are in the context of gut microbiomes not oral communities.

**We have acknowledged the reviewer's comments and rephrased the discussion to be more cautious and less speculative.**

 7. "These commensal oral bacteria may become opportunistic pathogens in immune-compromised states." please cite

**Idris A, Hasnain SZ, Huat LZ, Koh D. Human diseases, immunity and the oral microbiota—Insights gained from metagenomic studies. Oral Science International. 2017 Jul;14(2):27–32.**

****

**Cited in-text as well.**

 8. Fig 3- legends should indicate that a-b were based on Unw-UniFrac, and that C on W-UniFrac - Please depict % variation on axes 

**Done.**

 9. It is not clear how the post-hoc Dun's test were conducted-0 is this still based on PERMANOVA?

**Post-hoc Dunn's test was conducted using an OTU table. Permanova does not conduct pairwise comparison hence Dunn's test was chosen to test for differences between people with different BMI status (underweight, normal, overweight). And the test revealed that underweight individuals had microbiomes that was significantly different from both normal and overweight individuals.**

 10. ****"The Kruskal-Wallis test generated by QIIME revealed no significant difference." This is not clear - compared to what? 

 12. QIIME is just a software with plugins to perform statistical analyses - it is not clear when authors are using qiime or R and how these platforms would yield different results. Please avoid jargon as "generated by QIIME"

**For 10 & 12, previously qiime and R yielded different results (one not significant, one significantly different) for oral microbiomes and BMI. Upon closer inspection we realized this happened because of mismatching samples and OTU table upon input to R. We checked the other sets of data and the qiime results matches with R. Changes addressed in Results section.**

 11. "There was a significant difference between waist circumference and saliva microbiome composition (weighted UniFrac, p-value = 0.022). ​​​​​" This is not clear - you mean differences between the microbiomes of subjects with different waist circumference?

**The above sentence has been rephrased to "There was a significant difference in the saliva microbiome of Temiar who had a healthy waist circumference compared to those with abdominal obesity."**

 13. "The saliva microbiome showed no significant difference between former smokers and never-smokers, suggesting perhaps the saliva microbiome may recover partially to an environment prior to smoking". This is unclear but also speculative. 

 The discussion is highly speculative and should be toned down, mainly as far as the hypotheses behind the differences reported.

 Authors also seem to interchangeably extrapolate hypothesis on oral microbiome changes with gut microbiome changes in the context of lifestyle and diet (e.g. Prevotella) although diet may also be linked to oral microbiome modulation, this relationship is less clear, and taxa such as Prevotella, abundant in both gut and oral cavity, cannot be assumed to be modulated by diet in both sites

**This comment has been well-received and edits have been done to Discussion section.**

****

**TQ.**
